# Effect of Quantitative Nuclear Image Features on Recurrence of Ductal Carcinoma *In Situ* (DCIS) of the Breast

**DOI:** 10.4137/cin.s401

**Published:** 2008-03-01

**Authors:** David E. Axelrod, Naomi A. Miller, H. Lavina Lickley, Jin Qian, William A. Christens-Barry, Yan Yuan, Yuejiao Fu, Judith-Anne W. Chapman

**Affiliations:** 1 Department of Genetics and Cancer Institute of New Jersey, Rutgers—The State University of New Jersey, 604 Allison Road, Piscataway, NJ 08854-8082, U.S.A; 2 Department of Pathology, University Health Network and University of Toronto, 610 University Avenue, Toronto, Ontario, Canada. M5G 2M9; 3 Henrietta Banting Breast Cancer Centre, Women’s College Hospital, University of Toronto, 76 Grenville Street, 7th floor, Toronto, Ontario, Canada. M5S 1B2; 4 Department of Statistics and Actuarial Science, University of Waterloo, 200 University Avenue West, Waterloo, Ontario, Canada. N2L 3G1; 5 Equipoise Imaging LLC, 4009 St. Johns Lane, Ellicott City, MD 21042, U.S.A; 6 Department of Mathematics and Statistics, York University, 4700 Keele Street, Toronto, Ontario, Canada. M3J 1P3; 7 National Cancer Institute of Canada Clinical Trials Group, Queen’s University, 10 Stuart Street, Kingston, Ontario, Canada. K7L 3N6

**Keywords:** breast ductal carcinoma in situ, nuclear grade, image cytometry, discriminant analysis

## Abstract

**Background:**

Nuclear grade has been associated with breast DCIS recurrence and progression to invasive carcinoma; however, our previous study of a cohort of patients with breast DCIS did not find such an association with outcome. Fifty percent of patients had heterogeneous DCIS with more than one nuclear grade. The aim of the current study was to investigate the effect of quantitative nuclear features assessed with digital image analysis on ipsilateral DCIS recurrence.

**Methods:**

Hematoxylin and eosin stained slides for a cohort of 80 patients with primary breast DCIS were reviewed and two fields with representative grade (or grades) were identified by a Pathologist and simultaneously used for acquisition of digital images for each field. Van Nuys worst nuclear grade was assigned, as was predominant grade, and heterogeneous grading when present. Patients were grouped by heterogeneity of their nuclear grade: Group A: nuclear grade 1 only, nuclear grades 1 and 2, or nuclear grade 2 only (32 patients), Group B: nuclear grades 1, 2 and 3, or nuclear grades 2 and 3 (31 patients), Group 3: nuclear grade 3 only (17 patients). Nuclear fine structure was assessed by software which captured thirty-nine nuclear feature values describing nuclear morphometry, densitometry, and texture. Step-wise forward Cox regressions were performed with previous clinical and pathologic factors, and the new image analysis features.

**Results:**

Duplicate measurements were similar for 89.7% to 97.4% of assessed image features. The rate of correct classification of nuclear grading with digital image analysis features was similar in the two fields, and pooled assessment across both fields. In the pooled assessment, a discriminant function with one nuclear morphometric and one texture feature was significantly (p = 0.001) associated with nuclear grading, and provided correct jackknifed classification of a patient’s nuclear grade for Group A (78.1%), Group B (48.4%), and Group C (70.6%). The factors significantly associated with DCIS recurrence were those previously found, type of initial presentation (p = 0.03) and amount of parenchymal involvement (p = 0.05), along with the morphometry image feature of ellipticity (p = 0.04).

**Conclusion:**

Analysis of nuclear features measured by image cytometry may contribute to the classification and prognosis of breast DCIS patients with more than one nuclear grade.

## Introduction

The proportion of breast cancers currently being diagnosed in the *in situ* stage (ductal carcinoma in situ, DCIS) has been increasing with the increased use of mammographic screening and now accounts for up to about 25% of breast cancers diagnosed in some centers. Nuclear grade is a major determinant of therapeutic decisions although there is no single accepted grading system (Badve et al. 1998; Holland et al. 1994; Leonard and Swain, 2004; Leong et al. 2001; Silverstein et al. 1996; Silverstein, 2000; Sneige et al. 1999; Tavassoli, 1998; van Dongen et al. 1992; Wärnberg et al. 2002; Wärnberg et al. 1999).

Nuclear grade has been associated with DCIS recurrence (Badve et al. 1998; Burstein et al. 2004; Lagios et al. 1989) and progression to invasive carcinoma (Lagios et al. 1989; Kerilkowske et al. 2003). Based on such results, nuclear grade has been recommended for the primary stratification of DCIS (Schwarz, 1997). However, the association between nuclear grade and recurrence of DCIS or development of invasive carcinoma is not as clear in some studies in which as many as 50% of patients are reported to have more than one nuclear grade (Miller et al. 2001). In Miller et al. 2001 attempts were made to reflect and evaluate heterogeneity by evaluating both most dominant (most prominent) grade and worst grade; however, nuclear grading was not associated with clinical outcome. In this context, a single grade may not be sufficient to characterize a patient and this may affect the medical therapeutic decisions being made. It is important to recognize and account for heterogeneity of nuclear grade within each patient since as many as 50% of patients with DCIS may have more than one nuclear grade (Miller et al. 2001; Goldstein and Murphy, 1996).

It would be advantageous to have a method to obtain reliable quantitative data that could be objectively analyzed to assist with the classification of patients who have breast DCIS in which more than one nuclear grade is present. Computer-aided image analysis is such a method. Image analysis has been used to extract quantitative nuclear information useful for diagnosis of biopsy specimens of many tissues, including breast DCIS, and invasive breast (Kerlikowske et al. 2003;Carpenter et al. 1985; Dey et al. 2000; Frank et al. 2001; Hoque et al. 2001; Mariuzzi et al. 1996; Mommers et al. 2001; Susnik et al. 1995; Tuczek et al. 1996; Wolberg et al. 1995). However, these previous reports did not focus on evaluating information for individual nuclei in the classification of patients with mixed nuclear grades. We previously examined the role of image analysis of individual nuclei for the Miller et al. 2001 cohort with respect to the development of invasive breast cancer, and found that the reproducible, quantitated image analysis features were associated with development of invasive disease (Chapman et al. 2007) whereas nuclear grade was not. In that study, we determined that indications for particular image features across fields were not consistent when there was heterogeneous grading.

The purpose of this study was to determine, if after considering clinical and pathologic factors, whether nuclear features measured by digital image analysis of hematoxylin and eosin stained slides would be significantly associated with DCIS recurrence, and therefore could help pathologists classify patients whose DCIS was more likely to recur. The clinical cohort of patients whose DCIS exhibited substantive heterogeneity (Miller et al. 2001) was used for these investigations. We restricted the examinations to pooled assessments across fields, and an overall patient basis, for better per patient representation of nuclear features.

## Patients and Methods

### Patients and specimens

The DCIS patients whose specimens were used in this study were a subset of the 124 previously described in detail (Miller et al. 2001; Fish et al. 1998; Chapman et al. 2007). Of the 124, the cohort of 88 patients who underwent lumpectomy alone were used, as these patients had experienced most of the events: 17 of 19 recurrences of DCIS with median 5.0 years of follow-up. Of these 88 patients, three did not have slides available for assessment and for five patients the hematoxylin and eosin (H&E) stained slides resulted in images of poor quality, unsuitable for further evaluation. Digital images of the slides of the 80 study patients were acquired under the supervision of a breast pathologist (NM). Worst nuclear grade of 1, 2, or 3, by the Van Nuys system (Silverstein et al. 1996; Silverstein et al. 1995) was assigned by the pathologist by viewing the slides directly in a bright field microscope, yielding 1 patient with grade 1, 31 with grade 2, and 48 with grade 3 DCIS.

The focus for this study was an investigation of the effects of heterogeneity on the assessment of image analysis features. When more than one grade was present, predominant grade, and all grades were recorded for each of two representative fields for each patient. There were 32 patients in Group A, whose DCIS was nuclear grade 1 only (1 patient), grades 1 and grade 2 (8 patients), or grade 2 only (23 patients); 31 patients in Group B with grades 1, 2, and 3 (2 patients), or grades 2 and 3 (29 patients); and 17 patients in Group C with grade 3 only.

### Segmentation and preparation of image analysis data

As described above, DCIS heterogeneity was observed pathologically with H&E slides. Digital images of areas of DCIS identified by the pathologist were captured with a CCD camera, bright field microscope, desktop computer, and NIH-Image software, as described in detail in the Appendix. The experimental design involved imaging five ducts in one field, followed by imaging five ducts in a second field. Those fields demonstrated DCIS of the nuclear grade(s) recorded for that patient. The fields were present as two separate fields on one slide or one field on each of two slides. Thus, ten or more images in different regions of the slides(s) were captured for each patient, 20 or more nuclei per image were segmented resulting typically in 200 nuclei analyzed for each of the 80 patients. The focus for these investigations are the two fields with about 100 nuclei assessed per field and the pooled data for both fields, to yield an overall assessment for a patient. A blank field was subtracted from each captured image. Images were printed and nuclei labeled with an index number to avoid duplication. Contrast enhanced images were viewed on a 17 inch monitor and enlarged 4:1. These changes did not affect the values extracted from the saved image. Each nuclear region of interest was segmented by an operator using a computer mouse. The operator was blinded to the grade of the specimen determined by the pathologist. All nuclear images were segmented by a single person (DA). The reproducibility of feature values extracted by operator guided segmentation is discussed in the Results section. Some image feature calculations and the merging of all nuclear image feature data to per field and per patient attribution were accomplished with StatView v 5.01 (Brain Power, Calabasas, CA, USA) software.

### Image analysis features

For each nucleus, 39 features were determined in three categories. (i) Morphometry: area, perimeter, ellipse major axis, ellipse minor axis, ellipticity (major axis/minor axis), shape form factor (4 X pi X area/perimeter squared), and roundness b (4 X area/pi X ellipticity squared) (Russ, 1990). (ii) Densitometry: mean density, standard deviation of density, modal density, minimum density, maximum density, sum density (mean density X area, used instead of I.O.D. of NIH-Image), range density. (iii) Markovian texture features (Haralick et al. 1973; Pressman, 1976) were calculated from the co-occurrence matrix of pixel densities with a step size of 2. They were angular second moment, contrast, correlation, variance, inverse difference moment, sum average, sum variance (corrected from Pressman, 1976), difference average, difference variance, initial entropy, final entropy, entropy, sum entropy, difference entropy, coefficient of variation, peak transition probability, diagonal variance, diagonal moment, second diagonal moment, product moment, and triangular symmetry. Additional texture features, calculated from the binned histogram of pixel gray scale values, included histogram mean, histogram variance, histogram skewness, and histogram kurtosis. Similar features have previously been used to classify breast ductal carcinoma in situ specimens (Frank et al. 2001; Hoque et al. 2001).

### Prognostic factors

The clinical factors recorded on these patients were age (in years) and type of presentation (mammographic, clinically palpable, bloody nipple discharge). The histologic factors previously evaluated (Miller et al. 2001) were maximum DCIS size (cm), percentage of parenchyma involved with DCIS (<10%, 10%–50%, >50%), predominant architecture (0—cribriform/micropapillary/other, 1—solid), worst architecture (0—cribriform/ micropapillary/other, 1—solid), nuclear grade [by the Van Nuys Classification system worst (nuclear grade 1, 2, 3); also, predominant (nuclear grade 1, 2, 3)] and heterogeneous nuclear grading (Group A, Group B, Group C)], necrosis [none, confluent (comedo-like)], calcification (none, crystalline/ amorphous), measured margin (zero margin, <1 mm, 1–5 mm, >5 mm), presence of uninvolved intervening duct (not assessable, no, yes), Van Nuys Prognostic Index. In addition, 39 nuclear image features were determined for about 200 nuclei per patient.

For each patient, the image data were pooled across i) all nuclei in a field (2 assessments), and ii) all nuclei for a patient (1 assessment) to yield a summary feature value [adjusted mean = mean/ (standard error of the mean)], for each of the 39 image features for nuclei of the 3 different assessments per patient: 2 fields, 1 overall. In addition, grading discriminant classification functions, that are weighted combinations of image features, described below in the Analysis section, were assessed as prognostic factors.

Three different assessments, corresponding to the 3 different ways of pooling the image analysis feature data, were performed to examine the effects of DCIS heterogeneity on apparent associations with clinical outcome. In other contexts, investigations have been restricted to single ducts, fields, or pooled per person assessments without an examination of replicability.

### Events

Recurrence of ipsilateral DCIS made more than 90 days after the initial surgery was designated as an event. There were no developments of contralateral DCIS. There were no deaths from breast cancer, or another cause, in this group of patients over the study period.

### Statistical analysis

Statistical analyses were performed with BMDP PC Dynamic Version 7.0 (same as BMDP-XP, Statistical Solutions, Sagua, MA, USA). Analyses included for each image feature and each patient, 1) Levene’s tests for equality of variance between fields for each person and between people, 2) use of the mean/S.E.M. of image features on a field and patient basis due to highly significant evidence against assumption of equal variances, 3) forward step-wise Fisher linear discriminant analyses, using an entry p-value of p = 0.05, and 4) jackknifed (leave-one-out) classification of patients to find the number of patients who were correctly classified by the discriminant functions.

The histologic, clinical, and image analysis factors were assessed with respect to whether they were associated with ipsilateral DCIS recurrence. Univariate assessments were with Kaplan-Meier plots and the Wilcoxon (Peto-Prentice) test statistic (Prentice and Marek, 1979); for each image feature, standard image analysis cut-points at the means of the data were utilized after confirmation that the data were approximately symmetric.

Multivariate assessments utilized continuous data where possible, and were with Cox forward stepwise regressions, using the likelihood ratio criterion (~χ^2^_(1),_ p ≤ 0.05) as the test statistic to determine if a factor would be added to the model. Since we had no knowledge of which of the image analysis features assessed would best reflect a patient’s DCIS, or the extent to which differences in image features might relate to prognosis, we performed 3 sets of multivariate analyses, corresponding to the 3 generations of image feature factors per patient: per 2 fields, 1 pooled across 2 fields.

## Results

### Suitability of H&E stained slides for image analysis

Archival H&E slides were satisfactory for image analysis for the majority of this DCIS cohort (Chapman et al. 2007). Only 5.7% (5/88) patients had slides that were of too poor quality.

### Reproducibility of segmentation and image measurements

All nuclear regions of interest were segmented manually by the same operator (DA). Reproducibility of manual segmentation was determined by repeatedly segmenting the same nucleus (CV = 3.4%, n = 150). In order to determine the reproducibility of extracted feature values, independent measurements were made of the same nucleus in images captured at different times. Ten or more nuclei, identified from images of specimens of seven patients, were segmented at two different times without knowledge of the previously segmented region of interest, or of the extracted feature values. The differences between the pairs of measurements of the same nucleus were determined with a two-tailed t-test. (The null hypothesis was that the differences were equal to zero, df = n-1, at the 0.05 level of significance). The percent of feature values that were not statistically different in duplicate measurements ranged from 89.7% to 97.4% among nuclei of the seven patients. Further, there were no statistically significant differences for 23 of the 39 feature values in 7/7 pairs of images, and 37 of 39 feature values in at least 5/7 pairs of images. Among the features whose values had minimal differences in all pairs of images there were two that were selected by discriminant analysis, the morphological feature ellipse minor axis and the texture feature peak transition probability.

### Classification of patients by image analysis

In order to take into account patients with mixed grades, patients were classified as defined above into Groups A, B, C, corresponding to low, intermediate and high grades. These grading groups were used in discriminant analyses for nuclei in field 1 (80 patients), field 2 (79 patients), and overall pooled across both fields (80 patients). The results of the three discriminant analyses are provided in Table 1. In each instance (field 1, field 2, and overall across both fields), there were image features significantly associated with the grading classifications, p < 0.001 for each. The discriminant function for the first field included one morphological feature reflecting the size of nuclei (minor ellipse axis) and one texture feature reflecting the arrangement of DNA in the nucleus (sum entropy). Discriminant analysis of the second field included one morphological feature (perimeter), one densitometric feature (range density) and one texture feature (angular second moment). The analyses for both fields indicated one morphometric feature (minor ellipse axis) and one texture feature (peak transition probability). Different image analysis features were obtained for the first field, second field, and both fields. Correct classification of the nuclear grading with the image features for each field was respectively, 65%, 67.1% and 65.0%, in Table 1.

**Figure 1:**
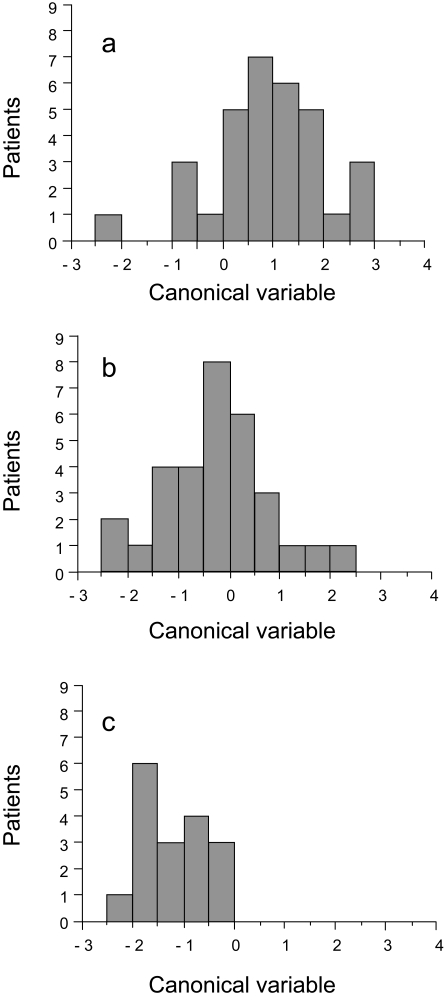
Distribution of patients between groups. **a**, Group A; **b**, Group B; **c**, Group C. The value of the discriminant function for each patient is determined by a weighted combination of image features significantly associated (p < 0.001) with the characteristics of the grading groups. Factors that dealt with a larger minor ellipse axis, indicative of a rounder nucleus, (p < 0.001) and lower peak transition probability, indicating more uniform nuclear staining, (p < 0.001) were associated with higher grading.

**Figure 2:**
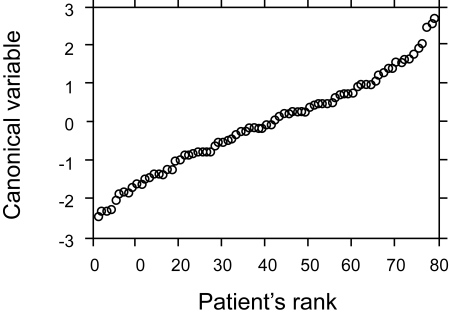
Distribution of all patients by rank according to the value of their canonical variable. There is a nearly continuous distribution of patients.

Discrimination using both fields would be most representative for a patient. A larger minor ellipse axis, indicative of a rounder nucleus, (p < 0.001) and lower peak transition probability, indicating more uniform nuclear staining, (p < 0.001) were associated with higher grading. Table 2 indicates the accuracy of image analysis classification by nuclear grading group. Image analysis features correctly classified 78.1% of patients in Group A (grade 1 and grade 2 nuclei), 48.4% in Group B (grade 2 and grade 3 nuclei), and 70.6% in Group C (grade 3 nuclei). The discriminant function was optimized to separate patients into the three nuclear grading groups. The distribution of patients within each of the three groups is illustrated in Figure 1. For each patient the canonical variable = 1.00475 X (minor ellipse axis) – 0.60149 X (peak transition probability) – 1.7285, where the canonical variable has zero mean and coefficients standardized by pooled within group variance, F-statistic = 12.304, df = 4,152, p < 0.001. Although the discriminant function was optimized to provide the best classification of patients within the three grading groups, there was considerable overlap between patients in the three grading groups. Figure 2 shows the discriminant function values ranked according to the value of the patient canonical variable; there is a nearly continuous distribution.

**Figure 3:**
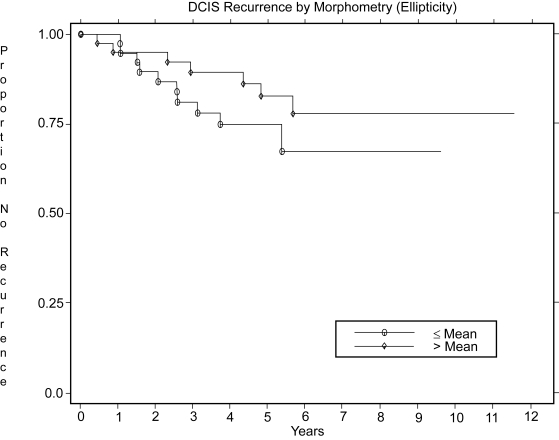
Kaplan-Meier plot of image feature significantly associated with recurrence of DCIS: Morphometry (ellipticity), p = 0.04. The recurrence rates at 5 years are 25% for patients with Ellipticity less than or equal to the mean, and 17% for those with Ellipticity greater than the mean; the numbers of patients remaining at risk at 5 years are respectively, 23 and 24.

Table 3 indicates the factors significantly (p ≤ 0.05) associated with DCIS recurrence in the multivariate analyses based on data for the 2 fields and overall pooled assessments. Each model contains both clinical and image features; however, the image features differ. The overall pooled data would represent the best available summary for the patients. The factors significantly associated with recurrence of DCIS, were those previously found by Miller et al. 2001, type of initial presentation (p = 0.03), and amount of parenchymal involvement (p = 0.05), along with the morphometry image feature ellipticity, p = 0.04. Figure 3 shows the Kaplan-Meier plot for the image feature; smaller ellipticity (less elongated and more rounded nuclei) was associated with higher DCIS recurrence Van Nuys nuclear grade, predominant grade, and the grading discriminant function were not significantly associated with recurrence of ipsilateral DCIS.

## Discussion

Nuclear grade, or all grades when there was more than one grade, was reported for each patient by the pathologist according to the Van Nuys system (Holland et al. 1994; Silverstein et al. 1995). This system has been compared to other systems that have been proposed to predict development of infiltrating carcinoma (Badve et al. 1998; Leong et al. 2001; Douglas-Jones et al. 1996). Several of these grading systems have recently been reviewed (Leonard and Swain, 2004). The greatest consistency among pathologists seems to be obtained with systems that are based, in large part, on nuclear grade (Morrow et al. 2002) and a consensus conference on classification of DCIS recommended that DCIS should be stratified primarily by nuclear grade (Schwartz, 1997). Our results indicate that image analysis can provide a reproducible quantitative description of nuclei for this purpose since duplicate measurements were similar for 89.7% to 97.4% of features assessed.

We investigated the ability of 39 image features describing nuclear morphology (size and shape), densitometry (amount of stain), and texture (arrangement of DNA) to quantitatively discriminate tumors with pathologically determined nuclear grade. The most representative grading for a patient utilized data obtained for nuclei in both fields. Features included in the discriminant function were one whose value was determined by the size and shape of the nuclei (minor ellipse axis) and one whose value was determined by the arrangement of DNA in the nuclei (peak transition probability). Similar rates of accurate classification of grade were obtained from the first field assessed (about 100 nuclei), the second field (about 100 nuclei), and both fields (about 200 nuclei), with correct classifications of respectively 65.0%, 67.1%, and 65.0% of the patients. However, the discriminant functions for the three situations differed by image features. Discrimination using both fields would be the most representative of the patient’s grading.

Discriminant analysis has frequently been used to classify patients based on feature values determined by computer-aided image cytometry (Baak et al. 1991; Bartels, 1980; Patterson, 1995). The features selected and the weights assigned to each feature are readily interpretable. Further, we examined the relevance of the continuous discriminant functions here in a censored survival analysis framework.

In this study, image features were extracted from H&E stained slides rather than Feulgen stained slides. The Feulgen reaction is useful because it is specific for DNA, the intensity of the reaction is proportional to the amount of DNA, and nuclear regions of interest can be automatically segmented (Schulte et al. 1995). However, conventional H&E stained slides have also been used to extract morphometric and other nuclear feature values (Frank et al. 2001; Tuczek et al. 1996; Wolberg et al. 1995; Christen et al. 1993; Peinta and Coffey, 1991; Weyn et al. 2000). The possibility of extracting nuclear features from H&E stained slides offers several advantages. First, existing H&E stained slides available from pathology archives can be used without recovering the original paraffin blocks, recutting tissue sections, and staining the new slides with the Feulgen procedure; second, digital images of each microscopic field can be acquired by the image analysis system simultaneously with review by a pathologist viewing familiar H&E stained tissue. Although the density measurements extracted from H&E stained slides are not directly proportional to amount of DNA as they are for Feulgen stain, we have found that nuclear area is proportional to amount of DNA as determined by Feulgen (r = 0.886). Nevertheless, measurement of nuclear area in H&E stained specimens is not an adequate substitute for measurement of DNA ploidy as determined with the DNA-specific Feulgen stain. In the future, the labor intensive chore of manually segmenting nuclear regions of H&E stained slides might be overcome by acquiring color images and segmenting them with appropriate algorithms (Ferr-Roca et al. 1998; Latson et al. 2003). In our study only 5.7% of the archival slides had too poor stain quality for image analysis.

Patients with breast DCIS that had intermediate nuclear grades, and/or more than one grade, offer challenges to pathologists. Such patients may represent as much as 50% of DCIS patients (Miller et al. 2001). In this study computer-aided image analysis was used to assess a cohort of patients who presented with DCIS, many with intermediate or mixed grades. H&E stained slides were viewed by a pathologist who recorded the nuclear grades in each field and digital images were acquired simultaneously. Nuclear image feature values were extracted and patients classified by discriminant analysis using the pathologist’s grouping of patients to supervise the analysis. Patients were placed into three groups according to the grades assigned by the pathologist taking into account the large proportion of patients with intermediate and/or mixed grades. The discriminant function correctly classify 78.1% of patients with low nuclear grade, and 70.6% of patients with high nuclear grade. The same discriminant function had a poorer success rate of only 48.4% for the intermediate group. This suggests that the intermediate group could be subtyped by characteristics different than those separating the low and high grade groups. Such subtypes of the intermediate grade group might be relevant for therapeutic decisions.

Since high nuclear grade is one of the factors that has been associated with recurrence it might be expected that high nuclear grade would also be associated with recurrence within this cohort of patients. However, neither the highest nuclear grade, nor the most prominent nuclear grade, was associated with recurrence (Miller et al. 2001; Chapman et al. 2007). The large proportion of patients with mixed nuclear grades may have contributed to the lack of association. In order to take into account the patients with mixed grades, discriminant analysis used the pathologist’s nuclear grade(s) for each patient, the grouping of patients with mixed grades, and the quantitative nuclear feature values determined by image cytometry. A discriminant function was derived that optimized the classification of patients into grading groups. The value of the canonical variable was determined for each patient. The canonical variable was standardized across patient values to have a mean of zero and a standard deviation of one. The distribution of patient values by canonical variable appeared to be continuous rather than three discrete and separate groups. The discriminant function was not associated with ipsilateral DCIS recurrence, although it had been with some pooled assessments concerning the development of invasive breast cancer (Chapman et al. 2007). One of the image features included in the discriminant function was minor ellipse axis (p < 0.001); it is noteworthy that a related feature, ellipticity, was associated with DCIS recurrence (p = 0.04). Rounder nuclei were associated both with higher grade and DCIS recurrence. It should be noted that this association was found with image feature adjusted means, after accounting for the greater variability observed with high nuclear grade. The image features alone would not be expected to be sufficient for prognosis. However, the additional information, added to other pathologic and molecular features, may improve the prognostic classification of patients.

## Conclusion

A discriminant function was derived that optimized the classification of DCIS patients with mixed nuclear grades. The classification of patients into grading groups was significant (p < 0.001), although the separation of patient grading groups was not complete. Grouping patients with mixed nuclear grades and measuring their nuclear image features may contribute to their classification and prognosis.

## Figures and Tables

**Table 1:** Image features discriminating grading groups.

Field^a^	Image features	p-value^b^	Correct classification^c^
Field 1	Morphologic: minor ellipse axis	<0.001	65.0%
	Texture: sum entropy		
Field 2	Morphologic: perimeter	<0.001	67.1%
	Texture: angular second moment		
	Densitometric: range density		
Both fields	Morphometric: minor ellipse axis	<0.001	65.0%
	Texture: peak transition probability		

a Field 1 and Field 2 each had 5 different ducts.

b p-values are based on F-statistic of final discriminant models.

c Jackknifed (leave-one-out) classification.

**Table 2:** Classification of groups with image features.

Group	Patients in group	Number of patients classified into group			Percent correct
		A	B	C	
A	32	25	4	3	78.1
B	31	8	15	8	48.4
C	17	0	5	12	70.6
Total	80	33	24	23	65.0

**Notes:** Classification is based on about 200 nuclei per patient in two fields. Jackknifed (leave-one-out) assessment of classification was used.

**Table 3:** Clinical, histologic, and image analysis factors affecting DCIS recurrence by image analysis assessment.

Field 1		Field 2	
Factors^a^	p-value	Factors^a^	p-value
Texture (Histogram mean)	0.01	Densitometry (Range density)	<0.001
Initial presentation	0.01	Measured margin	<0.001
Parenchymal involvement	0.02	Densitometry (Sum density)	0.02
Architecture	0.05	Van Nuys Prognostic Index	0.04
** Both fields – Overall**			
Morphometry (Ellipticity)	p = 0.04		
Initial presentation	p = 0.03		
Parenchymal involvement	p = 0.05		

a Factors significantly (p ≤ 0.05) associated with DCIS recurrence, in the order entered into the step-wise forward Cox regression models.

## Appendix

### Image analysis system

#### Hardware

A custom image cytometry system was assembled which consisted of a CCD camera attached to a bright field microscope and linked to a desktop computer with a frame grabber card. Images of nuclei were acquired and stored as follows: hematoxylin and eosin stained slides were viewed with a bright field microscope (Wild model M20), 40X N.A. 0.75 objective, 1.25X phototube, 530–590 nm band pass green filter, detected with an 8 bit monochrome CCD camera (Sonyo model VDC3874) connected to a video monitor (RCA TC1112) and a frame grabber card (60 HZ Data Translation Quick Capture model DT2255) in a desktop computer (Apple Macintosh model IIci, 12 MB RAM, 80 MB hard disk), and stored as uncompressed TIFF files on removable media (Zip 100 disks). Ten frames were averaged and acquired using NIH-Image software (v. 1.57, written by Wayne Rasband, obtained from the internet by anonymous FTP). Each TIFF formatted image was 640 × 480 pixels, with 256 gray levels. The resulting pixel images were isotropic, with an effective size of 0.25 microns × 0.25 microns. Segregated nuclear images were of modest resolution, typically containing 800 to 1600 square pixels. Sizes were calibrated with a B&L stage micrometer. Optical density of pixel gray values was standardized and camera response calibrated with a set of neutral density filters (50, 25, and 12.5% transmission).

#### Software

NIH-Image v.1.62b34-Arnv software (modified from http://rsb.info.nih.gov/) and StatView v. 5.01 statistical package (BrainPower, Calabasas, CA, USA) were used to measure and calculate DNA densitometric and nuclear morphometric features using a Mac G4 computer. TextureCalc v. 1.1ax, software (written by W. C.-B.) was used to rebin 256 gray levels into 8 intervals and to calculate texture features from the Markovian gray level co-occurrence matrices. Programs written in SAS release 6.12 for the Macintosh (SAS Institute, Inc., Cary, NC, USA) were used to format data and BMDP PC Dynamic Version 7.0 (Statistical Solutions, Sagua, MA, USA) statistical software was used for discriminant analysis.
